# INTERPERSONAL STRESSORS, AWARENESS OF AGE-RELATED CHANGES, AND ARTHRITIS ON DAILY MEMORY FUNCTION

**DOI:** 10.1093/geroni/igae098.0550

**Published:** 2024-12-31

**Authors:** Shalini Mukherjee, Shevaun Neupert

**Affiliations:** North Carolina State University, Raleigh, North Carolina, United States; North Carolina State University, Raleigh, North Carolina, United States

## Abstract

The current study examined the interaction between daily awareness of age-related changes (AARC) losses and daily interpersonal stressors in predicting daily memory failures among 440 older adults in the U.S. (50-85 years) with and without arthritis. Participants reported their arthritis diagnosis on the first day of the study and then completed daily AARC and stressor questionnaires for the subsequent 13 days. Multilevel modeling analyses revealed a 3-way interaction for Daily Interpersonal Stressors X Daily AARC Losses X Arthritis. Among people with arthritis, high daily AARC losses were consistently associated with a high level of memory failures, irrespective of exposure to interpersonal stressors. However, individuals with arthritis who experienced low levels of AARC losses showed a significant increase in daily memory failures when exposed to interpersonal stressors. The findings suggest that interpersonal stressors are particularly powerful for individuals with arthritis when they experience low levels of AARC losses. Conversely, for those without arthritis, interpersonal stressors are linked to an increase in memory failures, regardless of AARC losses. Further research is recommended to understand the underlying mechanisms of the interaction and develop targeted interventions to improve memory function in individuals with arthritis, as well as those without the condition who are experiencing stress. Overall, the study’s findings have significant implications for clinical practice and highlight the importance of addressing psychosocial factors in the management of arthritis and other age-related conditions.

